# Functional dynamics of primate cortico-striatal networks during volitional movements

**DOI:** 10.3389/fnsys.2014.00027

**Published:** 2014-03-10

**Authors:** Lucas Santos, Ioan Opris, Robert Hampson, Dwayne W. Godwin, Greg Gerhardt, Samuel Deadwyler

**Affiliations:** ^1^Department of Physiology and Pharmacology, Wake Forest University Medical SchoolWinston-Salem, NC, USA; ^2^Department of Neurobiology and Anatomy, Wake Forest University Medical SchoolWinston-Salem, NC, USA; ^3^Department of Neurobiology and Neurology, University of KentuckyLexington, KY, USA

**Keywords:** motor cortex, motorstriatal circuit, mechanism of parkinson's disease, topographic of motor striatal circuit, simultaneous recording of sensorimotor striatal circuit with drugs

## Abstract

The motor cortex and dorsal striatum (caudate nucleus and putamen) are key regions in motor processing but the interface between the cortex and striatum is not well understood. While dorsal striatum integrates information from multiple brain regions to shape motor learning and habit formation, the disruption of cortico-striatal circuits compromises the functionality of these circuits resulting in a multitude of neurologic disorders, including Parkinson's disease. To better understand the modulation of the cortico-striatal circuits we recorded simultaneously single neuron activity from four brain regions, primary motor, and sensory cortices, together with the rostral and caudal segments of the putamen in rhesus monkeys performing a visual motor task. Results show that spatial and temporal-task related firing relationships between these cortico-striatal circuit regions were modified by the independent administration of the two drugs (cocaine and baclofen). Spatial tuning and correlated firing of neurons from motor cortex and putamen were severely disrupted by cocaine and baclofen on correct trials, while the two drugs have dramatically decreased the functional connectivity of the motor cortical-striatal network. These findings provide insight into the modulation of cortical-striatal firing related to movement with implications for therapeutic approaches to Parkinson's disease and related disorders.

## Introduction

The motor cortex and dorsal striatum (caudate nucleus and putamen) of the brain are the key regions in motor processing (Alexander et al., 1990; Kalaska et al., 1997). Although the cortical control of movement is well documented (Kalaska et al., 1997; Taylor et al., 2002; Carmena et al., 2003; Andersen et al., 2004; Donoghue et al., 2005; Lebedev et al., 2008; Nicolelis and Lebedev, 2009), the interaction between cortex and striatum (Opris et al., 2013) is also relevant. This is mainly because the striatum receives input from topographic projections (that are responsible for turning relevant behaviors on and off, depending on the behavioral context), from roughly 90% of the cortex (Bolam et al., 2000; Graybiel, 2004; DeLong and Wichmann, 2007). While dorsal striatum integrates information from multiple brain regions (Wall et al., 2013) to shape motor learning and habit formation, the interface between cortex and striatum is not well understood.

An important reason we looked into this aspect stems from the fact that disruption of cortico-striatal circuits compromises the functionality of these circuits, resulting in a multitude of neurologic disorders, including Parkinson's disease (PD). PD is one of the most devastating neurodegenerative disorders, with no cure and limited treatment (Camicioli, 1993; Graybiel, 2004). PD is characterized by symptoms that include tremors and muscle stiffness with strong cortical-basal beta (15–30 Hz) oscillation in human and animal models (Leventhal et al., 2012). Dopamine (DA) depletion in the substantia nigra, which innervates the striatum, is the main cause of PD (Kish et al., 1988). Thus, current treatments aim to replace dopamine (e.g., levodopa/L-DOPA) or to apply deep brain stimulation, which can also be effective (Fahn et al., 2004; Siddiqui et al., 2010; Deuschl et al., 2013).

Here, we report electrophysiological recordings of coordinated activity in this motor circuit while the system is engaged in a motor task and employing pharmacological treatment to perturb the system and to reveal novel aspects of the motor circuit function.

An intriguing observation concerns the indirect influence of cocaine on the action mechanism in the striatal circuit through the inhibition of dopamine (DA) reuptake, which modulates the DA transporter, thereby prolonging levels of DA in the synapse (Wise, 1984). It has been hypothesized that this motor striatal circuit gate may enact our behavior and enable the habits. In addition, cocaine effectively acts on this sensorimotor mechanism through the D1-like dopamine receptors in the stratum via gene expression (Willuhn and Steiner, 2008). Therefore, administration of psycho-stimulants (such as cocaine) may cause a state of hyper-dopaminergic intracellular tone within the motor circuitry in which a variety of disorders, such as Parkinson's disease, Huntington's disease, Alzheimer's disease, dementia with Lowy bodies, etc., have been demonstrated (Piggott et al., 1999; Willuhn and Steiner, 2006; Vohora and Bhowmik, 2012). By the same token, although there is no effective treatment available for such disorders in the motor circuitry, baclofen, a gamma-aminobyturic acid B receptor (GABA-B) agonist, has been widely used in clinical settings for muscle relaxation, treatment of spasticity in spinal cord injury, stroke (Rekand et al., 2012), and for cerebral palsy in children (Morton et al., 2011). This leads us to hypothesize that, as both cocaine and baclofen influence the motor cortex and striatum (basal ganglia), both would provide a reliable approach to studying the functional relationship of this cortical-striatal circuit. Thus, our findings may contribute to establishing innovative approaches to the sufferers of movement disorders.

## Methods

All animal procedures were reviewed and approved by the Institutional Animal Care and Use Committee of Wake Forest University, in accordance with US Department of Agriculture, International Association for the Assessment and Accreditation of Laboratory Animal Care and National Institutes of Health guidelines. The animals were housed individually in cages that were twice the area (12.4 cu ft) typically provided for rhesus monkeys. The temperature, humidity, and light controlled conditions were 22 ± 2°C, 50 ± 30% relative humidity, and a 12-h light/dark cycle, respectively. Appropriate food, water, treats and vitamin supplements were provided, and animals were given access to environmental enrichments, such as approved toys, swings, perches, and mirrors or music to promote psychological well-being in accordance with the Association for Assessment and Accreditation of Laboratory Animal Care (AAALAC), 7th Edition, 1996, National Research Council, U.S.

**Figure 1:**
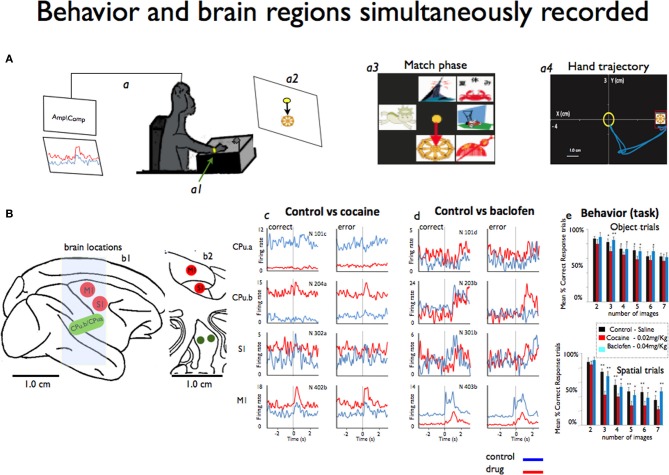
**Simultaneous recording of behavior and neural activity from four different brain regions. (A)** A monkey performs the delayed match-to-sample (DMS) task while sitting at the behavioral apparatus attached to the recording brain **(a)** activity (Amp/Comp) and the position of the right arm is tracked via an illuminated UV- fluorescent reflector **(a1)**. The display screen shows an image sample **(a2)** and in more detail the match phase in which the sample phase image was presented with distracting images **(a3)**. Also shows an example of the x-y hand trajectories **(a4)** during target selection. **(B)** The sagittal view of the four brain regions **(b1)** recorded from during the behavioral task: M1: motor cortex, S1: somatosensory cortex, CPu.a and CPu.b: respective rostral and medial putamen. Also shows the coronal view **(b2)** in depth of the same locations. **(c)** Mean firing rates from the same animal displayed as peri-event histograms (PEHs) ±2 s prior to the onset of match phase (0.0 s shown in **A**) on correct vs. error DMS trials recorded simultaneously, in cocaine and baclofen (respectively **c**,**d**) from each of the four regions shown in **(B)** in sessions in which either cocaine or baclofen were administered midway through. In **(e)** we shown the behavior task performance during object and spatial trials (up and down) respect to the image numbers. See video in supplementary information for example of the cortico-striatal recordings.

Four monkeys (rhesus, Macaca mulatta) were trained to perform (Figure 1) a delayed match-to-sample (DMS) visual motor task, (Opris et al., 2009; Porrino et al., 2013) for juice rewards to a criterion performance level that was stable for at least 1 year. Efforts were made to use the minimal number of animals in this study. All animals were under the care and supervision of veterinarians.

This study was designed to demonstrate neuronal interactions between sensorimotor cortex and the dorsal striatum (e.g., two segments of putamen: one on the medial part and the other 5 mm rostral from the center of the recording cylinder) that were simultaneously accessed for the first time in NHPs. It was based on 10 experiments/sessions performed in four NHPs weighing about 8–11 kg each. Each animal was implanted with two recording cylinders (Crist Instruments) implanted over the targeted brain areas: one over the motor cortex (central sulcus) and another one overlaying the prefrontal cortex. The experimental procedure took place through the caudal (motor) cylinder. Most analyses used for this data have been previously published in detail (Hampson et al., 2004; Porrino et al., 2005; Deadwyler et al., 2007; Opris et al., 2009; Santos et al., 2012). During recording, a customized 16 amp tetrode micro-device was positioned in the chamber with their guide tubes (~100 um diameter) protruding through the dura mater, and the tetrodes were individually lowered to the recording sites (Santos et al., 2012). The tetrode wires (17–25 μm), with an impedance of about 1.5 MΩ, were plated to reduce the resistive component of impedance to ~0.1 MΩ. The major advantage of using this recording method is (a) the size of recording probes inserted into the brain is ~10 times smaller (~100 μm) than the traditional probes for recording in deep brain regions, (b) it also allows to record more units with better signal-to-noise ratio, and simultaneously from several regions. All recordings and animal performance in the delayed match-to-sample (DMS) task were well-characterized and documented, (Hampson et al., 2004; Porrino et al., 2005; Deadwyler et al., 2007; Opris et al., 2009). Hand coordinates (X, Y, and velocity) of the movement positions associated with task events (Figure 1A) were recorded simultaneously with the cells and synchronized on the same clock, using previously established methodology for cell and behavioral recordings (Nicolelis et al., 2003; Hampson et al., 2004; Dzirasa et al., 2006; Fetz, 2007; Santos et al., 2008, 2012).

### Behavior

All animals sat in a primate chair in front of a display screen and moved a cursor with their right arm to perform the visually guided responses required by the DMS task (Figure 1A) to obtain a juice reward on each trial(Deadwyler and Hampson, 2004). The position of the right arm was tracked via an illuminated UV-fluorescent reflector (Figures 1A,a1) affixed to the wrist, digitized, and displayed as a large yellow cursor on the projection screen. The animals were trained to a stable baseline performance level of 70–80% (of ~200 trials), with different numbers (2–7) of images and delay duration reflecting task difficulty (Hampson et al., 2009). Each trial was initiated by the animal placing the cursor inside a 3-in diameter circular yellow outline (“start ring”) in the center of the screen. Response to the start ring produced one of two visually distinct target images positioned at random locations on the screen. Movement of the cursor into the go target images for at least 500 msec, within 2.0 s of the presentation of a response (correct or error), produced a squirt (100 ms) of juice. Incorrect responses (e.g., failure to respond to the proper target within 2.0 s or touching the not-target image with the cursor during the 5.0 s timeout interval) were not rewarded and were followed by a 5.0 s delay with the screen blanked and no trials (Hampson et al., 2004). The trials were presented randomly, separated by 3–10 s inter-trial intervals. Each animal was recorded once a week.

### Recordings and data analysis

We used a 64-multichannel acquisition processor (MAP Spike Sorter by Plexon, Inc. Dallas, TX) for the recordings, and principal component analysis (PCA) was performed in 2D/3D via standard parametric multivariate analyses of variance (MANOVA) (Nicolelis et al., 2003) for single neuronal cluster validation (Davies and Holdsworth, 1979; Wheeler, 1999). The MAP (and MAP cluster) provided further amplification and band pass filtering (500 Hz–5 KHz) to these signals. After a stage of analog-to-digital conversion, the signals were routed to DSP boards, each of which contained four 40 KHz digital signal processors (DSP, Motorola 56002). The DSP boards also provided inputs for sampling digital pulses, generated to monitor the animal's behavior during the execution of tasks and to synchronize performance with brain signals. NeuroExplorer (NexTechnologies, Littleton, MA) software and MATLAB (The Mathworks, Inc.) routine codes were used to rectify the data analysis (Santos et al., 2012). In each session, the DMS task trials were split into two parts: the first part of 30 min recording without drugs (control session) and the second part of 1 h of recording after we injected the drug (0.02 mg/kg *i.v* of cocaine and 0.2 mg/kg of baclofen). Only cells that responded to the behavior session (control) were included in the analysis.

In this study we examined cortico-striatal circuitry using a novel approach of simultaneous recordings of single neurons during behavior in two (A1, A2) nonhuman primates (NHPs). Two NHPs were recorded during i.v administration of cocaine and two NHP during administration of baclofen while performing the visuomotor delayed-match-to-sample (DMS) task shown Figure 1A (Hampson et al., 2004; Opris, 2013). Although these regions are well understood with regard to movement disorders, the interrelationship of the responses in these areas by means of simultaneous recording is less known. Implementation of the novel tetrode microdevice (Santos et al., 2012) allowed simultaneous recording of the targeted locations (Figures 1B,b1,b2) during the DMS task (Hampson et al., 2010; Santos et al., 2012).

In each DMS task trial, a sample image was selected while neural activity was recorded in motor and somatosensory cortices, as well as in the two segments (rostral and medial) of striatum/putamen. Supplementary information (movie of cell recording samples) shows illustrative samples for typical motor and striatum neurons recorded. The x-y coordinates of the moving cursor were also recorded with the hand trajectories of movements in the same task during the match phase to correlate firing with hand position as displayed respectively in Figures 1A,a3,a4.

Individual spikes were used to construct peri-event histograms (PEHs) and raster displays for single neurons (Opris et al., 2013), and two dimensional joint peri-stimulus time histograms (JPSTH; (Vaadia et al., 1995) to quantify the synchronization and the cross-correlation of two selected neurons (x motor; y striatum) in this network as shown in Figure 4. JPSTHs and cross-correlation histograms (CCHs) are generated using a shift predictor algorithm built into NeuroExplorer (http://www.neuroexplorer.com/).

#### Tuning plots

**Figure 2:**
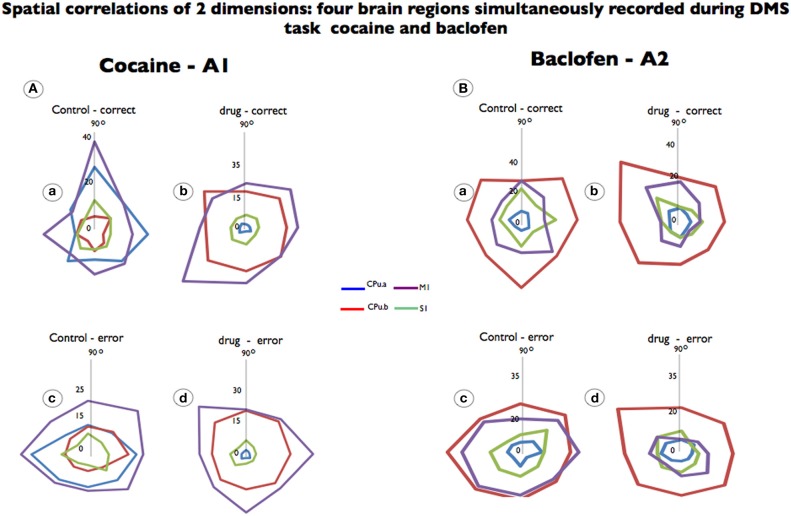
**Spatial tuning of firing from each brain region simultaneously recorded during DMS task performance.** Recordings are shown for the same neurons recorded prior to drug administration (control) and after cocaine or baclofen injection (Figure 1B). **(A)** Shows color-coded directional spatial tuning plots of the task-related discharges of cells recorded in the rostromedial striatum (CPu.a and CPu.b) and the primary motor and sensory cortices (S1 and M1) at the indicated Cartesian (x-y) hand coordinates calculated for the eight horizontal planes (120 cm^2^) during target selection. The neurons were recorded under two conditions: control (saline) and cocaine (0.02 mg/kg). M1 and CPu.b tuning were preserved but changed by cocaine administration while S1 and CPu.a were eliminated. **(B)** Shows a similar display for neurons recorded from the same four regions during the task following injections of baclofen (0.2 mg/kg). Data are presented as averages, to which firing rate changes are indicated by the vertical axis in the middle (0) of each graphic.

For each analyzed neuron, firing on the same trials was aligned to presentation of the match target position selected. Directionality was assigned according to 8 different “clock” directions corresponding to the location of the match image around the periphery of the screen, yielding 0°, 45°, 90°, 135°, 180°, 225°, 270°, 315°, and 360° movement directions (from center of screen). Mean firing rate after match presentation but immediately prior to match response (i.e., 0.0–1.0 s) for each response position was calculated and represented in polar coordinates as a tuning plot (Opris et al., 2013). The directional bias for a given cell was revealed by the response locations with the highest mean firing rate *and* by the direction of tuning vectors computed during the match epoch (Figure 2, polar plots).

#### Statistical analyses

Standard (Z) scores of increased firing rates relative to nonevent baseline values are calculated for individual cells for each task event. Firing rate is analyzed in 50 ms bins for ±2 s relative to time of initiation (0 s) task events. Only neurons with firing rates significantly elevated from that in pre-event baseline (−2 to 0 s) period are included for analysis. Differences in mean firing rate and CCHs between neuron spikes of cortical and striatal cell pairs are assessed using ANOVA and paired *t*-test.

## Results

A total of *n* = 263 single neurons in five sessions—were recorded from striatum (*n* = 113), motor cortex (*n* = 90) somatosensory cortex (*n* = 60). The cortical cells have fired in the 1–10 Hz range, while the interneurons in the 10–40 Hz range. We did not run our analysis focusing on a specific group of neurons as we were more interested in their modulation during the DMS behavior.

Cocaine strongly inhibited the firing of striatal neurons in the rostral segment (CPu.a) with 95% on correct trials than when using saline as a control (Cocaine: 2.5 ± 0.16 Hz vs. Control; 24.3 ± 0.34 Hz mean ± s.e.m.) and on 76% on error trials (Cocaine: 2.9 ± 0.14 Hz vs. Control; 22.3 ± 0.47 Hz) in the animal shown in Figures 1B,d. We observed opposite responses from the medial putaminal segment (CPu.b) topographically orientated to the motor cortex, recorded in the control vs. cocaine sessions. The firing rate significantly increased by 24 vs. 42% (Cocaine; 12.01 ± 0.37 Hz vs. Control; 2.8 ± 0.10 Hz and Cocaine; 11.5 ± 0.32 Hz vs. Control; 4.8 ± 0.10 Hz), as shown in Figures 1B,d *P* < 0.001, One-Way ANOVA (Sakurai and Takahashi, 2006; Opris et al., 2011) relative to the correct and error responses of both conditions.

A vast body of literature has demonstrated the heterogeneity of the striatum, in that it is behaviorally segregated into limbic, sensory and motor divisions (Middleton and Strick, 2000; Takada et al., 2001), which is confirmed by these results mainly through rostral and medial (CPu.a and CPu.b) neuronal relationships with respect to motor cortex (Figures 1B,c,d).

Neurons in motor cortex (M1) increased their firing rate by ~39 vs. 27% as a result of cocaine administration during correct and error responses (Figures 1B,c; *P* < 0.0001 ANOVA). There was no statistical difference in the S1 neurons recorded under the influence of cocaine: (2.6 ± 0.16) Hz, (3.0 ± 0.17) Hz on correct or (2.3 ± 0.14) Hz; (2.7 ± 0.12) Hz on error trails and response was stronger on error trials under cocaine influence than on correct trials (*P* < 0.9 and 0.1 s.e.m.; ANOVA).

The same regions were recorded under the influence of 0.2 mg/kg of baclofen (Figures 1B,d) in two different animals. There were no significant changes in the firing of neurons following baclofen administration for the two regions of the striatum/putamen (CPu.a, CPu.b), except for correct responses (*P* < 0.0001; ANOVA). Conversely, the firing rate of S1 and M1 neurons decreased significantly (*P* < 0.0001; ANOVA) for both correct and error trails. Stronger effects of cocaine were observed for M1 that shows an increased firing with 44% (*P* < 0.001; ANOVA), and 59% decreased firing rate for both error vs. correct responses under the influence of baclofen (*P* < 0.001; ANOVA). It is clear that each neuronal region has its own preferential firing direction regardless of motor or putamen areas recorded, as it has been previously pointed out (Georgopoulos and Ashe, 1991). Both drugs affected the behavior performance on object or spatial trials (Figures 1B,e).

We analyzed the preferential discharge rate of the same neurons for 8 movement directions (Figure 2) related to the visuomotor DMS task. Cocaine changed the preferential discharge for neurons firing to targets in each location on the screen. While striatal neurons (CPu.a) have a preferential discharge at 90°, the same neurons decreased preferential discharge following cocaine injection and firing rate was reduced by 96% to 1.2 Hz (Figures 2A,a,b; *p* = 0.0001; ANOVA) eliminating significant differences between correct and error responses. By the same token S1 neurons, which fired preferentially at 90°, lose their tuning completely (Figures 2A,b) and decreased firing rate. However, the neurons from the brain regions functionally related to movement, like the medial striatal segment (CPu.b), have enhanced firing rate (from 7.2 Hz at 270° to 23.0 Hz at 135°) and the motor cortex (M1) in which preferential discharged was at 90° during control (19.3 Hz) tilted 45° (225°) is firing at 34.6 Hz (Figures 2A,a,b; *p* = 0.001 ANOVA). Although the preferred direction changed for the recorded neurons under control vs. drug in correct trials, it was not statistical significant for those neurons when compared in error (Figures 2A,c,d) responses.

Cell firing preferred directions were also examined following baclofen administration in which firing changes were observed with respect to preferred direction and firing rate. Although we observed that cells simultaneously recorded from the so-called motor striatal circuit changed their preferential discharges, such preferred direction was independent from those recorded with cocaine. The only statistically significant effects of firing to position were observed for motor cortex (16 ± 0.73 Hz vs. 8.3 ± 0.64 Hz; *p* < 0.0001; ANOVA) in which responses in error control increased the firing rate, while the opposite was observed in drug error trials (Figures 2B,c,d).

**Figure 3:**
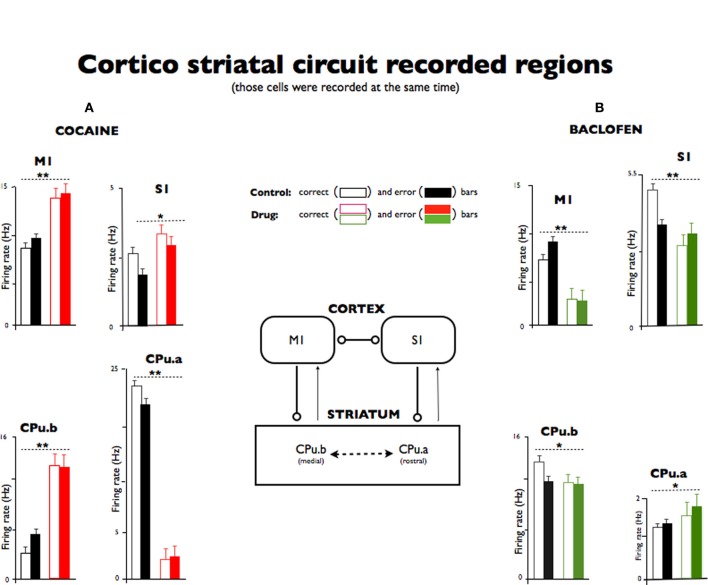
**Simultaneous recorded neurons in sensory and motor cortices and striatum under different task conditions and following cocaine and baclofen administration. (A)** Cortex (S1, M1) and striatal (CPu.a and CPu.b) neuron firing rates during correct vs. error responses (open and black filled bars) recorded in control (saline) vs. cocaine treated trials (red open and red filled bars). Cocaine strongly increased the firing rate in the motor cortex and in the striatal medial segment (M1, CPu.b) and to a lesser degree in S1, and strongly depressed activity in rostral striatal neurons (CPu.a). **(B)** Neurons recorded in the same four areas from another animal in control (black) vs. baclofen (green) trials segregated by the same open filled bar scheme of A with green bars representing firing on baclofen trials. Neurons in both cortical areas (S1, M1) and the medial striatum (CPu.b) decreased firing following exposure to baclofen while the rostral segment of striatum (CPu.a) exhibited slightly increased firing. No statistical significance was found between correct vs. error responses neither for cocaine nor for baclofen in this study. A schematic of corticostriatal neurons representing recorded regions is to illustrate the mechanism of the loop circuit. All error bars indicated s.e.m. (^*^P < 0.05; ^**^P < 0.001).

Figure 3 shows recordings in the same task in which cocaine increased firing in M1, CPu.b, S1 and depressed activity in the rostral striatum (CPu.a, Figure 3A), but there was no significant change with respect to correct vs. error trials. The results of baclofen injection allowed us to observe changes in the motor-striatal circuitry during synaptic suppression has enhanced GABAergic action in which this circuit has been implicated (Albin et al., 1989; Helmich et al., 2012). Motor and sensory cortices, as well as the striatal medial segment (CPu.b^*^) exhibited inhibition (Figure 3B^**^), while the rostral striatal segment (CPu.a^*^) showed enhanced activity, exhibiting the intrinsic interactive nature of this network.

**Figure 4:**
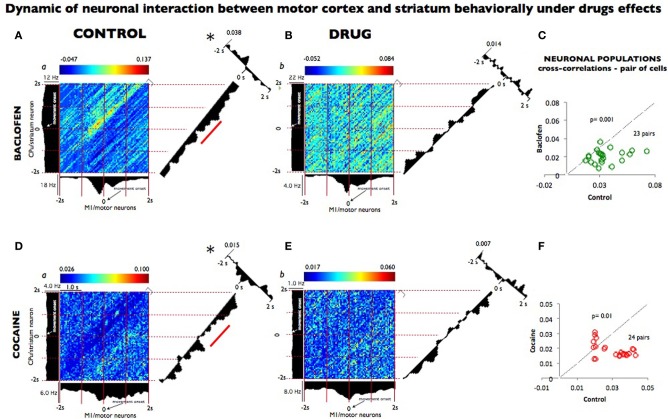
**Dynamic relationship of neuronal pairs from motor cortex and striatum modulated during control vs. drug conditions. (A)** Joint peri-stimulus time histograms (JPSTHs) constructed (Vaadia et al., 1995) from pairs of neurons in the motor cortex and in the striatum, which were respectively recorded during nondrug (control) conditions for 0.5 h in 53 trials. The color-coded pixels of the matrix represent the normalized correlation coefficient of neuronal peak. Each bin size corresponds to the correlation of the two neuron pairs as a function of coincidence in time. The (blue/red) color scales represent the mini and max integration, respectively, which is proportional to synaptic strength. The time windows are depicted on the right corner of each matrix. The firing rate (Hz) for each neuron pair is shown on the left and top of the respective motor striatum histograms. The diagonals (representing the coincident firing) show the 2 s window period sampled and binned at 50 ms from both neurons during the match phase in the DMS task (Grid intervals, 1 s). Each bin size corresponds to the degree of correlation between the two-neuron pairs within the same time bins as shown in the color scale above each plot (a). The diagonal of the JPSTH is the coincidence histogram, indicating the temporal evolution of the correlation in the color-coded “raw” JPSTH matrix between the plotted neuron pairs (abscissa, motor, and ordinate striatum). A bin size of 50 ms was used to calculate correlations along both angles. Both diagonals show a clear modulation that indicated connectivity between the motor cortex and the striatum, which disappeared completely in the presence of baclofen shown in **(B)** (1 h, 104 trials). On the right angle to the coincidence histogram, one-dimensional cross-correlogram (CCH; 2 seconds bin-wise normalized) depicts the correlation between the two neuron regions; the synaptic strength of this relationship is on the top left. **(C)** The population plots for JPSTH diagonal cells (*n* = 23) pairs, from which samples **(A,B)** were used, under control performance and following baclofen administration. In **(D)**, the same JPSTH analysis was applied to pairs of cells consisting of a motor neuron (5,282 and 10,653 spikes); and striatum neuron (3,959 and 2,301 spikes) simultaneously recorded from control (a) conditions (0.5 h, 77 trials) and following cocaine (b) administration (1 h, 152 trials). The CCH became poorer in the neuron pairs recorded after cocaine injection **(E)** compared with the control **(D)**, as indicated by the loss of inhibition noted by the diagonal matrix and in CCH modulations. **(F)** The population plots for the CCH diagonal of the neuronal pairs cells *n* = 24 following cocaine administration (**D** and **E**). Temporal modulation changes in the majority of the neuron populations recorded in baclofen were statistically greater (*p* = 0.001) than the ones recorded in cocaine (*p* < 0.01; ANOVA), though in both pairs sampled, such changes were significant^*^ (*p* = 0.001) compared to control recordings.

In order to effectively quantify the relationship within the cortico-striatal circuit we computed the temporal dynamics between the spike trains of neurons from motor cortex and striatal neurons recorded after baclofen and cocaine injection in both animals performing the DMS task. Individual spikes were used to construct two dimensional Joint peri-stimulus time histograms (JPSTH) to quantify the correlation and peak cross correlation of two selected neurons (x motor; y striatum) in this network as shown in Figures 4 (A,B,D,E, respectively for baclofen and cocaine cell pairs). Data were sampled in 50 ms bins (Vaadia et al., 1995) for both types of correlations. Figures 4A,B show a strong functional connectivity on the JPSTH diagonal and on the cross correlation between M1 and striatum, which was reduced following administration of baclofen. The same effect was observed in the cross-correlations of multiple pairs of cells analyzed under the same conditions (Figure 4C; *P* = 0001, *n* = 23 pairs; One-Way ANOVA). The same method of analysis (Vaadia et al., 1995) was used to test a similar hypothesis after cocaine administration, with respect to the functional connection between motor cortex and striatum during the DMS task. Indeed, this question has been addressed previously (Albin et al., 1989; Camicioli, 1993; Graybiel, 2004), but has not been examined in behaving animals where those regions were recorded simultaneously. Furthermore, in the motor-striatal neuron pairs, opposite effects were observed in the cross-correlations under baclofen vs. cocaine administration (Figures 4D–F, *P* < 0.0001, ANOVA). Both, the diagonal of the JPSTH matrix, as well as the cross correlations between neuron pairs in each area were disrupted by cocaine and baclofen, whose compromised connection is implied in a number of movement disorders.

## Discussion

Our results show for the first time single cell recordings of cortical and striatal brain areas simultaneously observed in freely behaving NHPs performing a visuomotor task, in which connectivity changed spatially and temporally as a result of the baclofen and cocaine drugs used.

The above results indicate a functional and temporal relationship between the sensorimotor cortex and putamen/striatum in behaving monkeys. The cortical-striate integrating mechanism where multiple circuits overlap is functionally relevant for the control of sensorimotor integrated behavior (de Wit et al., 2012). The two drugs used, cocaine and baclofen, play critical roles in modulating dopamine reuptake in the striatum and in the underlying signaling pathway for the active behavior mechanism of cortical inhibition in humans, respectively (Lyons et al., 1996; McDonnell et al., 2006). Although these approaches have been reported, they usually are not tested in the same behavioral context, nor by observing simultaneous interaction by means of accessing the neural firing in cortex and striatum in primates performing a task.

Moreover, it has been previously demonstrated that dopamine modulates neural firing in the NHP striatum and sensorimotor cortical regions (Gerhardt et al., 1995; Cragg et al., 2002; Wilson et al., 2009; Rekand et al., 2012). Cocaine has been used as a potent tool for studying modulation of dopaminergic (Lyons et al., 1996; Tropea et al., 2011) circuits. In addition, the GABA_B_ agonist baclofen, whose mechanism of action has been widely used in clinical settings for muscle relaxation, spasticity in spinal cord injury, stroke (Rekand et al., 2012), and, in children, for cerebral palsy (Morton et al., 2011).

The first observation revealed by our results is that cocaine influenced the system broadly, significantly affecting the putamen and motor cortex. The striatal medial segment (CPu.b) topographically orientated to the motor cortex enhanced its activity, whereas the rostral segment (CPu.a) had its firing rate reduced in the same recording session (Figures 1B,c). Alternatively, baclofen has a reversed (inhibition) and stronger effect than that observed with cocaine. Such characteristics resemble the interesting multimodal features (Andersen, 1997) suggested for sensorimotor-striatal circuitry.

In Figure 2, a similar observation is noted, wherein cocaine produced changes in the neurons' preferential direction, as did baclofen, but in addition, cocaine's effects caused a stronger firing-rate reduction in the sensorial neurons as well as in the rostral/CPu.a, a limbic segment.

Cocaine modulates the action of D1 dopamine receptors, associated with c-fos gene regulation in the striatum (Graybiel et al., 1990; Gerfen et al., 1995). Therefore, it has been suggested that cocaine encodes the transcription of motor skill learning (Willuhn and Steiner, 2008). Dopamine dysfunction or deficiency in the striatum is the major clinical observation in Parkinsonism (Khan et al., 2002). The influence of cocaine in the cortico-striatal circuitry affected the whole population of neurons analyzed. For example, we observed a difference in the rostral vs. medial (CPu.a vs. CPu.b) striatal neurons, with the rostral decreasing its firing rate by 96% whereas the medial striatal cells behaving inversely, enhancing firing rate by 50% during the neurons' preferential discharge (Figure 2A). Moreover, the connectivity-based segregation of the striatum concerns the rostral region as more associative with limbic functions, and therefore more related to psychiatric disorders, whereas the medial region has been regarded as more related to the motor cortex, being affected in motion/movement disorders (Alexander et al., 1990; Bohanna et al., 2011).

The current study has demonstrated not only the connection between the striatum and sensorimotor pathways, but it also shows for the first time such functional connection being active in a behavioral paradigm. Moreover, for a long time, the striatum has been considered a motor center almost exclusively (Middleton and Strick, 2000). However, our results show that an injection of 0.2 mg/kg of baclofen in the middle of a recording session produces change in response different from that observed by an injection of cocaine (Figure 3B). Baclofen acted even more strongly than cocaine on the disruption of the motor striatal temporal connection (Figure 4C) in neuronal population during the DMS task. Furthermore, the clinical and research relevance of cortical-striatal communications for movement disorders, including Huntington's disease, Lowy body dementia, and Parkinson's disease, has been pointed out elsewhere (Anderson and Reiner, 1990; Chase and Oh, 2000; Graybiel, 2004). Taking Parkinsonism as an example, the core of its neuronal dysfunction is in the striatum, the secondary consequence of dopamine depletion of neurons in the substantia nigra. In this context, the data presented here indicates that the multimodal circuit regions in which about 80–90% of all cortical projections send their output is to the striatum (Bolam et al., 2000).

One may question whether the observed effects might depend on the increased release of norepinephrine (NE) or serotonin (5-HT) or dopamine (DA), especially in the cerebral cortex. Recent results imply that dopamine and serotonin exerts a significant role in movement control by modulating the synaptic inputs of cortical (Ilic et al., 2012), striatal (Mathur and Lovinger, 2012a,b) and both pallidal segments and exerts a significant role in movement control (Kita et al., 2007). This depends on the spot since serotonin has a dynamic relationship with other transmitters and stimulation one spot can activate or inhibit specific neurons. On the other hand, motor skill acquisition (Lange et al., 2007, p.2251) may be modulated by the central NE-innervated alpha(1)-adrenergic receptor system that serves to co-excite or enhance signaling in several monoaminergic brain regions involved in movement and motor activity (Stone et al., 2003).

In summary, our findings suggest that neurons within the cortical-striatal network play different roles under the influence of cocaine and baclofen. Spatio-temporal modulation within this circuit illustrates how movement is encoded via the activity of neuronal assemblies (McHaffie et al., 2005; Feingold et al., 2012; Wymbs et al., 2012). These findings contribute to a better understanding of the functional interplay between elements of the cortical-striatal circuit. Because this circuitry is specifically impaired in Parkinson's disease and other movement disorders, a better grasp of the dynamic relationships within this circuit will assist in devising novel treatments in the future. Thus, understanding these network dynamics will assist in the development of neuro-prosthetic devices that will incorporate knowledge of subcortical as well as cortical structures in the coordination of motor commands.

Altogether, these results are consistent with previous works (Deadwyler and Hampson, 1995; Chapin and Nicolelis, 1996, 1999) regarding the functionality of these brain structures, therefore the novelty in our report is summarized by the following: (i) the simultaneous recording of cortical and striatal neurons during behavior; (ii) pharmacological modulation by agents that demonstrate the unique role of dopaminergic and GABAergic neurotransmission during the DMS task. Thus, it may shed further.

### Conflict of interest statement

The authors declare that the research was conducted in the absence of any commercial or financial relationships that could be construed as a potential conflict of interest.
